# All anti-CD20 monoclonal antibodies have similar efficacy and safety risks: Yes

**DOI:** 10.1177/13524585221108294

**Published:** 2022-09-20

**Authors:** Bruce AC Cree

**Affiliations:** Weill Institute for Neurosciences, Department of Neurology, University of California San Francisco, San Francisco, CA 94158, USA

The remarkable feature of monoclonal antibodies (MAbs) is target specificity. MAbs recognize specific molecular epitopes and seldom cross-react with other antigens. MAbs developed for multiple sclerosis (MS) treatment directed against CD20 recognize either neighboring or overlapping protein epitopes. These anti-CD20 MAbs deplete B cells through complement-dependent and antibody-dependent cellular cytotoxicity (ADCC). Glycoengineering of the FC region influences the relative contributions of these two processes. Thus, rituximab and ofatumumab deplete B cells primarily through complement fixation, whereas ocrelizumab and ublituximab deplete B cells more through ADCC.^1^ The net effect of treatment with these antibodies is rapid B-cell depletion to undetectable levels in peripheral blood that is sustained by ongoing treatment. Because these MAbs have the same impact on depleting B cells, their clinical impact and side effects are very similar.

Ocrelizumab depletes B cells in peripheral blood such that by 2 weeks post-treatment B cells are no longer detectable.^2^ Rituximab also results in rapid, near-total B-cell depletion 2 weeks after treatment.^3^ In a phase 2 study of ublituximab, B-cell counts were reduced by 97%, 24 hours after the first infusion, and by 4 weeks, B-cell depletion was reduced by >99% from baseline.^4^ Ofatumumab reduces B cells slightly less rapidly than the other MAbs. By 2 weeks, 82% of ofatumumab study participants had nearly undetectable peripheral B-cell counts, and by 12 weeks, 98% of participants had undetectable B cells.^5^ The difference between ofatumumab and infused MAbs is presumed to be due to the larger drug doses that can be administered intravenously compared to subcutaneously. All four MAbs efficiently maintain B cell depletion without reconstitution. Therefore, although the initial rates of depletion may differ slightly, the depth and maintenance of depletion appear to be common to all four treatments. Although it is conceivable that the rates of depletion might influence efficacy to some extent in some patients, after 12 weeks of treatment, such potential differences would no longer be relevant.

Given that B-cell depletion is efficiently achieved by all four MAbs, it seems likely that clinical efficacy will be similar. A major challenge to assessing efficacy across these products is the absence of head-to-head data. Cross-trial comparisons in multiple sclerosis are notoriously difficult to interpret largely because of the remarkable variability of the populations under study. Nonetheless, some of the commonly used efficacy assessments account for treatment effects based on a reference group such as the annualized relapse rate (ARR). The definition of MS relapse is well standardized in modern MS clinical trials. However, the choice of comparator influences the ARR ratio, and because not all trials used the same comparator, the comparison of the ARR ratios across trials is limited. Nonetheless, the ASCLEPIOS trials with ofatumumab and the ULTIMATE trials^6^ with ublituximab used teriflunomide as the same active comparator. Furthermore, both studies used twinned clinical trial designs with non-overlapping MS centers but identical protocols to ensure that observations made in one study would be replicated in a second independent dataset. The ARR ratios for ofatumumab versus teriflunomide were 0.49 and 0.42 for the ASCLEPIOS studies and for ublituximab versus teriflunomide were 0.51 and 0.42, respectively. Although it is formally possible that these studies showed strikingly similar ARR ratios due to chance, it seems more likely that their similarity is due to a shared therapeutic benefit in preventing relapses. Adding to this argument is the observation that the ARR ratio for ocrelizumab compared to thrice-weekly interferon beta-1a was 0.54 and 0.53 in the OPERA trials. All three drugs have remarkably similar effects in reducing the risk of clinical relapse because all products work equally well to deplete B cells, their common mechanism of action. Similar arguments can be made for other frequently observed medically relevant events such as gadolinium-diethylene-triamine penta-acetic acid (DPTA) T1 lesion or T2 lesion formation. Phase 3 clinical trials with rituximab were not conducted, and therefore data on ARR ratios for this product cannot easily be compared to the other three anti-CD20 MAbs.

Skeptics of the above argument could point to differences in disability worsening that at first glance appear to differ across studies. The ofatumumab clinical trials showed a statistically significant effect on confirmed disability progression (CDP); however, the ublituximab studies failed to show a statistically significant effect. Nonetheless, the magnitude of effect on 6-month CDP was nearly identical across the two studies: for ofatumumab, the hazard ratio (HR) was 0.68 (*p* = 0.01) and for ublituximab, the HR was 0.66 (*p* = NS). The event rates for CDP in the teriflunomide treatment arm differed across the studies with 4.8% versus 12.0% of teriflunomide-treated participants experiencing CDP in the ULTIMATE and ASCELEPIOS studies, respectively. Lower than expected CDP rates in the comparator arm and a much smaller study size (ASCLEPIOS enrolled 1882 participants, whereas ULTIMATE enrolled 1089) could underlie the differences in statistical significance for CDP across these studies. That ublituximab impacts disability is supported by the analysis of confirmed disability improvement that showed a HR of 2.03 (95% confidence interval (CI): 1.27, 3.25) favoring ublituximab. Finally, the HR for ocrelizumab versus thrice-weekly interferon beta-1a for 6-month CDP was 0.6. (*p* = 0.003), a result very similar to that for ofatumumab although the trials used different comparators. Data for an effect of rituximab on CDP are not available.

Safety concerns for anti-CD20 MAbs are generally shared although there are some important differences. Three of the products (rituximab, ocrelizumab, ublituximab) are infused intravenously and are associated with infusion reactions, whereas ofatumumab is self-injected and therefore is associated with injection reactions rather than infusion reactions. Furthermore, the need for diphenhydramine as a pre-medication to prevent infusion reactions depends on the infused MAb. The risk of infections, including opportunistic infections, also appears to be similar across these products and is directly linked to B-cell depletion. Finally, vaccination responses to Covid-19 RNA-based vaccines are probably similarly suppressed across all products although such data for ublituximab are not yet available.

In summary, anti-CD20 MAbs exert their therapeutic benefit through a common mechanism of action: the robust and sustained depletion of B cells. Phase 3 data for three of the four MAbs (ocrelizumab, ofatumumab, ublituximab) show strikingly similar effects on clinical and radiographic measures of disease activity. Furthermore, ocrelizumab and ofatumumab have similar effects on CDP, whereas ublituximab’s effect, although not statistically significant, showed a similar magnitude. Phase 3 data for rituximab are not available; however, the widespread clinical use of this product in Sweden^7^ is consistent with a clinical benefit that may be comparable to other products. Therefore, difference in clinical use of these medications will be based on routes of administration, duration of infusion, need for concomitant pre-medications, and patient and provider access to treatment.
